# Prenatal perfluorooctanoic acid exposure and glutathione s-transferase *T1/M1* genotypes and their association with atopic dermatitis at 2 years of age

**DOI:** 10.1371/journal.pone.0210708

**Published:** 2019-01-16

**Authors:** Hui-Ju Wen, Shu-Li Wang, Pau-Chung Chen, Yue Leon Guo

**Affiliations:** 1 National Institute of Environmental Health Sciences, National Health Research Institutes, Miaoli, Taiwan; 2 Department of Public Health, National Defense Medical Center, Taipei, Taiwan; 3 Institute of Occupational Medicine and Industrial Hygiene, National Taiwan University College of Public Health, Taipei, Taiwan; 4 Department of Environmental and Occupational Medicine, National Taiwan University (NTU) College of Medicine and NTU Hospital, Taipei, Taiwan; 5 Department of Public Health, National Taiwan University College of Public Health, Taipei, Taiwan; University of Cincinnati, UNITED STATES

## Abstract

**Background:**

Perfluoroalkyl substance (PFAS) exposure was found associated with atopic diseases. Atopic dermatitis (AD) is a childhood skin disorder. However, the effect of interaction between PFASs and glutathione S-transferase (*GST*) *T1/M1* genotype on AD remains unclear.

**Objective:**

To investigate the association between gene-environmental interaction and childhood AD using a birth cohort study.

**Methods:**

From 2001 to 2005, 1,264 mother–newborn pairs were recruited from eight Taiwanese maternity hospitals. PFAS levels and Genotypes were analysed from cord blood. Information on children’s health status including AD occurrence was obtained via phone interviews at 6 months and 2 years. Cord plasma concentrations of nine PFASs were measured via ultra-high performance liquid chromatography/tandem mass spectrometry. *GSTT1/M1* was genotyped (null/present) via polymerase chain reaction. Environment-gene interaction effects on AD were assessed using multiple logistic regression analysis.

**Results:**

Overall, 839 mother–newborn pairs completed all measurements. The prevalence of ever having physician-diagnosed AD by 2 years of age was 5.4%. Among PFASs, perfluorooctanoic acid (PFOA) was positively associated with AD adjusted for potential confounders. After grouping PFOA levels into three groups: undetected, below and above the median in those with detected, children in above the median group who had the *GSTT1-*null, or *GSTM1*-null genotype exhibited a higher odds ratio for AD (OR [95%CI] = 3.45 [1.26–9.99] and 2.92 [1.12–7.91], respectively) as compared to the undetected group.

**Conclusions:**

Our data demonstrated that in-utero PFOA exposure with *GSTT1/M1* null genotype were associated with AD. Minimizing early-life PFAS exposure may help against AD development, especially in genetically susceptible individuals.

## Introduction

Perfluoroalkyl and polyfluoroalkyl substances (PFASs) are widespread and persistent synthetic chemicals in the environment and humans. PFASs are composed of highly stable carbon-fluorine bonds and provide high chemical and thermal stability, durability, and strength [1]. They are widely applied in many products used in daily life including clothing, food packaging, carpets, furniture, and fire-fighting foams [2]. The health concerns for PFAS exposure are owing to their bioaccumulation and persistence [3].

Atopic dermatitis (AD) is a common childhood chronic skin inflammation disorder that has profound effects on the quality of life of the affected children and their families, especially in those with severe symptoms [4]. In Taiwan, the prevalence of physician-diagnosed AD among school-age children increased from 1.57% in 1995–96 to 2.79% in 2001[5] and among preschool-age children was 8.7% in 2008 [6]. AD is usually the first manifestation in the atopic march, which means that AD could be a significant predictor for other allergic disease such as asthma and allergic rhinitis [7]. Most AD symptoms occur in early life. Approximately half of children with AD have symptoms within the first 6 months of life and nearly 85% of affected children develop symptoms before the age of 5 years [8]. The identification of early risk factors of AD may allow for potential prevention against atopic disease development. Previously, we found environmental factors to be important components for development of AD [9]. Thus, we attended to define specific environmental factors considering genetics.

PFASs have been suggested to exhibit immunotoxicity from animal studies via altered cytokine production, inflammatory responses, and innate and adaptive immune responses [10]. PFASs have also been reported to associate with the development of atopic diseases in animals and humans. The immune responses in atopic diseases are found to be skewed toward a T-helper (Th) 2 phenotype with elevated levels of serum interleukin (IL)-4 and immunoglobulin E (IgE) [11]. Notably, Dong et al. found that perfluorooctane sulfonate (PFOS), one of the most common PFASs, was associated with increased secretion of IgE and Th2-type cytokines (IL-4 and IL-10) and decreased secretion of Th1-type cytokines (interferon [INF]-γ and IL-2) in mice [12]. Perfluorooctanoic acid (PFOA) was shown to be associated with increased IgE levels in a murine study [13]. In a case–control study, PFASs were shown to be associated with childhood asthma. Higher IgE concentrations were also found in children with higher PFAS levels [14]. However, the association between PFASs and childhood AD is still unclear and controversial. Okada et al. found that the risk of developing eczema decreased in children with lower prenatal perfluorotridecanoic acid exposure [15], whereas no association was found between PFAS exposure and AD in a study by Wang et al [16]. Thus, observation of a large number of children is necessary.

Genetic variation may play an important role in individual susceptibility to environmental pollutants. In the human body, glutathione S-transferase (GST) plays an essential role in chemical detoxification. *GST* genotypes are known to associate with the health effects of exposure to environmental pollutants including ambient air pollutants, smoke, metal, and pesticides. Chen et al. found that children carrying the *GSTM1*-null genotype had significant PM_2.5_-related increment in neutrophils and leukocytes in nasal lavage as determined by a longitudinal study of schoolchildren [17]. Wang et al. found that *GSTM1*-null and *GSTP1* Ile/Ile genotypes were associated with a significant increase in the risk of AD in children with prenatal smoke exposure [18]. Additionally, incense burning was reported to have a joint effect with *GSTT1* genotype and to associate with current asthma and wheezing in children [19].

Although genetic and environmental factors both likely contribute to the development of AD and PFAS exposure is known to be associated with atopic diseases, the effect of PFAS exposure and *GST* genotype on AD remains unclear. The current study aimed to investigate the effect of PFAS exposure and *GSTT1/M1* genotype on childhood AD from a 2-year follow-up birth cohort study.

## Materials and methods

### Study population and data acquisition

A longitudinal birth cohort study was conducted among pregnant women who had undergone prenatal examinations at eight selected private maternity hospitals located in seven areas, including one in Taipei, one in eastern Taiwan, and five in the south part of Taiwan. The newborns born after July 2001 were consecutively recruited [20]. After providing written informed consent, the pregnant women in their third trimester of gestation were asked to complete a structured questionnaire. Venous blood of the women and umbilical cord blood of newborns were obtained by a nurse. Blood specimens were centrifuged to obtain plasma and stored at −80°C until analysis. In total, 1,264 mother–newborn pairs were recruited between July 2001 and July 2005. Our protocols were approved by the National Cheng Kung University Hospital Institutional Review Board and the National Taiwan University Hospital Institutional Review Board.

### Data collection

Pregnant women were asked about their demographic characteristics, environmental factors at home (such as environmental tobacco smoke, cockroaches, incense burning, carpets, pets, or fungi on walls), family history of allergic diseases (atopic dermatitis, asthma, and allergic rhinitis), and neonate birth order. Maternal self-reported mental status during pregnancy was also included in the prenatal questionnaire. Newborn birth outcomes (gestational weeks, height, weight, and head circumference) were collected from hospital records by nurses.

Children were followed up via phone interview by well-trained interviewers at the ages of 6 and 24 months. After permission was obtained, mother or main caregiver was asked about the child’s growth situation, diet habit, health status, and environmental exposure. Children were considered as with- or without-AD according to the response to the question “Did your child ever have physician-diagnosed atopic dermatitis?” and “Did your child ever have symptoms of itching and scratching of the skin and have a rash characteristic in arm folds and behind the knees?” The answer of “Yes” to both questions in either of the two follow-up interviews was considered positive that the child had AD before 2 years of age.

### Analysis of PFASs

Cord plasma samples were sent to National Taiwan University for measurement of PFASs. Altogether, nine PFASs were analysed: perfluorohexanoic acid (PFHxA), perfluoroheptanoic acid (PFHpA), perfluorohexanesulfonic acid (PFHxS), PFOA, PFOS, perfluorononanoic acid (PFNA), perfluorodecanoic acid (PFDeA), perfluoroundecanoic acid (PFUnDA), and perfluorododecanoic acid (PFDoDA). The analytical method was as described in a previous study [21, 22]. The detection of PFASs in plasma were performed on an Agilent-1200 high performance liquid chromatography system (Agilent, Palo Alto, CA, USA) coupled with a triple-quadrupole mass spectrometer (Sciex API 4000, Applied Biosystems, Foster City, CA, USA). The limit of quantitation values (LOQ) for serum PFASs were 0.25, 0.28, 0.08, 0.45, 0.10, 0.11, 0.19, 0.13, and 0.07 ng/mL for PFHxA, PFHpA, PFHxS, PFOA, PFNA, PFOS, PFDeA, PFUA, and PFDoA, respectively. Participants' concentrations of PFASs below the detection limit were replaced by half-of-detection-limit values.

### Genotype determination

Genomic DNA was extracted from umbilical cord blood cells using standard genomic DNA extraction methods. The genotypes of *GSTT1/M1* were determined using polymerase chain reaction (PCR) assays as previously described [23]. GSTT1 and GSTM1 genotypes were classified as present type (heterozygous or homozygous genotype for the gene presence) and null type (homozygous deletion).

### Statistical analysis

JMP version 5.0.1 (SAS Institute Inc., Cary, NC, USA) was used to perform all statistical analyses. Geometric means of PFASs were calculated. Kruskal-Wallis tests was used to test for the differences in PFAS concentrations between children with and without AD after excluding outliers (S1 Table). Multiple logistic regression was also applied to test the association between PFAS exposure parameters and AD in children. Odds ratio (OR) and 95% confidence interval (95% CI) were used to assess the effects of PFAS exposure on AD. The stratified analysis by *GST* genotypes was performed to evaluate the effect of PFAS exposure and *GSTT1/M1* genotype on AD. *P* ≤ 0.05 was considered statistically significant.

## Results

**Fig 1:**
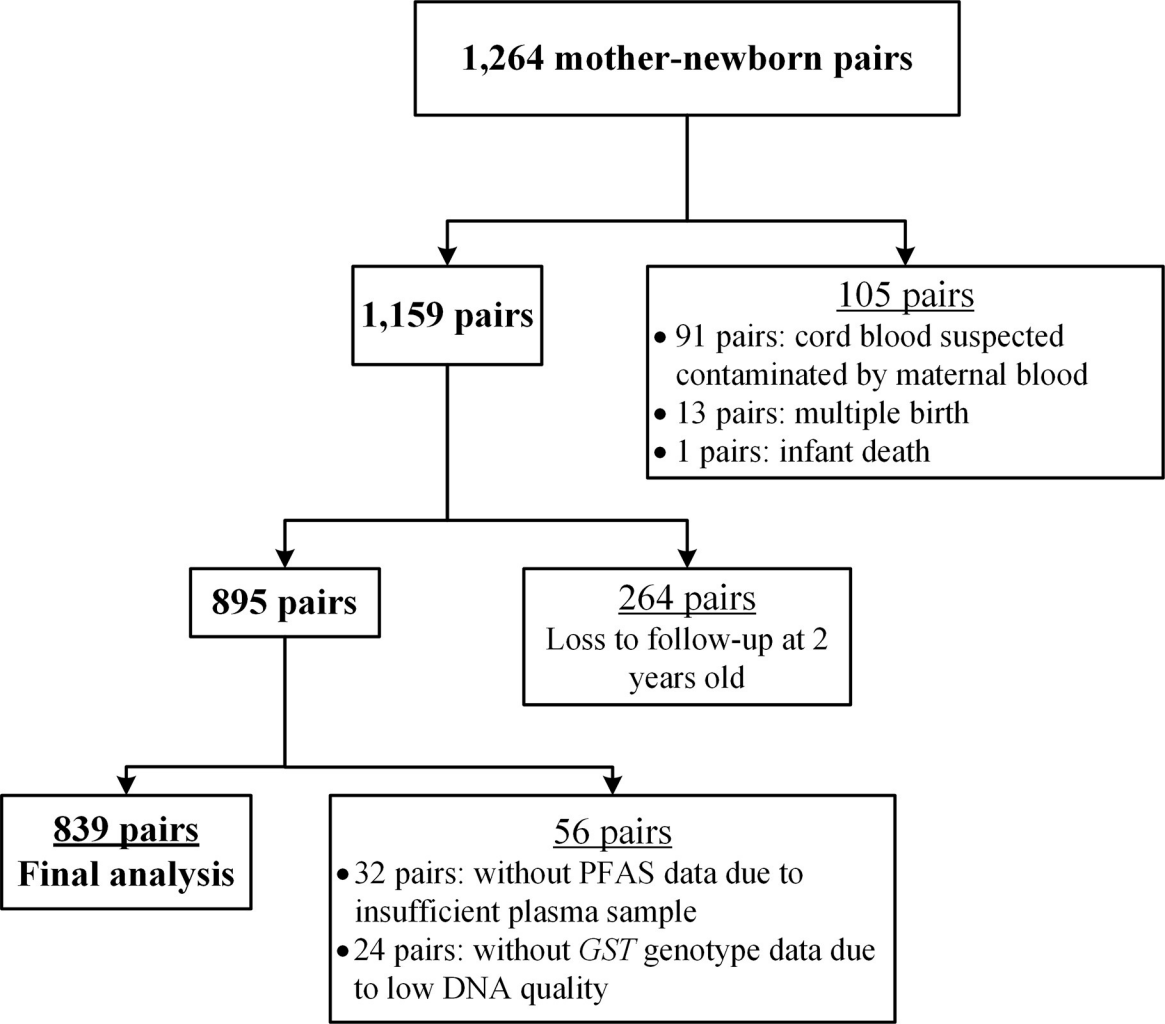
Flow chart of recruitment of mother-newborn pairs and data collection.

In total, 1,264 mother–newborn pairs who completed the questionnaire interview and specimen collection participated in the present study. After exclusion of 105 pairs because of suspected cord blood contamination by maternal blood (*N* = 91 pairs), multiple birth (*N* = 13 pairs), and infant death (*N* = 1 pair), 1,159 pairs were recruited in follow-up phone interviews. Among them, 264 pairs were lost to follow-up up to 2 years of age. We then excluded pairs without PFAS concentration (*N* = 32 pairs) or *GST* genotype (*N* = 24 pairs) data, resulting in 839 pairs recruited in the final analysis (Fig 1).

The prevalence of ever having physician-diagnosed AD in 2-year-old children was 5.4% (*N* = 45). For *GSTT1/M1* genotypes, the frequency of the null genotype was 49.8% (*N* = 418) for *GSTT1* and 56.3% (*N* = 471) for *GSTM1*. Table 1 demonstrates the characteristics of children and parents in children with and without AD. The children with and without AD did not differ significantly regarding birth weight, gestational weeks, sex, birth order, maternal age during pregnancy, paternal education, and family income. However, higher prevalence of breastfeeding and parental atopy was found in children with AD. The characteristics of children and parents between included pairs and excluded pairs are shown in S2 Table. No significant difference was found between included and excluded pairs in birth weight, gestational weeks, sex, birth order, maternal age during pregnancy, paternal education, family income, or parental atopy. However, included pairs had higher education levels of the mothers than excluded pairs.

**Table 1:** Characteristics of children and parents in children with and without AD.

Characteristic	Mean (SD) or *n* (%)		p-value^§^
	With AD (*n* = 45)	Without AD (*n* = 794)	
**Children**			
Birth weight (g)^¶^	3159.5 (427.0)	3101.7 (381.3)	0.677
Gestational age (weeks)^¶^	38.88 (1.23)	38.92 (1.20)	0.975
Sex			
Boy	25 (55.6)	399 (50.3)	0.489
Girl	20 (44.4)	395 (49.7)	
Birth order			
1^st^	24 (53.3)	334 (42.1)	0.316
2nd	17 (37.8)	273 (34.4)	
≥ 3rd	4 (8.9)	126 (15.9)	
Breastfeeding			
Yes	35 (77.8)	457 (57.6)	0.017
No	10 (22.2)	306 (38.5)	
**Parents**			
Maternal age at childbirth (year)^¶^	26.98 (4.29)	27.88 (4.71)	0.215
Maternal education			
≤ 9 years	3 (6.7)	81 (10.2)	0.719
10–12 years	26 (57.8)	448 (56.4)	
> 12 years	16 (35.6)	258 (32.5)	
Paternal education			
≤ 9 years	5 (11.1)	111 (14.0)	0.429
10–12 years	21 (46.7)	414 (52.1)	
> 12 years	19 (42.2)	258 (32.5)	
Family income (10^3^USD)			
<20	7 (15.6)	208 (26.2)	0.304
20–33.3	16 (35.6)	271 (34.1)	
≥33.3	18 (40.0)	270 (34.0)	
Maternal atopy			
Yes	15 (33.3)	151 (19.0)	0.020
No	30 (66.7)	641 (80.7)	
Paternal atopy			
Yes	16 (35.6)	154 (19.4)	0.009
No	29 (64.4)	638 (80.4)	
Smoking during pregnancy			
Yes	1 (2.2)	23 (2.9)	0.760
No	44 (97.8)	739 (93.1)	
ETS exposure during pregnancy			
Yes	25 (55.6)	412 (51.9)	0.666
No	17 (37.8)	322 (40.6)	

^¶^mean (SD).

^§^P value was calculated by Kruskal-Wallis tests for continues variables and χ2 test for categorical variables as compared between children with and without AD.

Some numbers do not add up to total n because of missing values.

Abbreviations: AD, atopic dermatitis; SD, standard deviation; USD, US dollars; ETS, environmental tobacco smoke.

Table 2 demonstrates the concentrations of nine PFASs in the cord plasma. All detection rates of PFASs were above 50% except PFHxA (3.33%), PFHpA (3.33%), and PFDeA (33.25%). These three PFASs were therefore excluded from further evaluation. A high correlation was found among PFNA, PFUnDA, and PFDoA as shown in S3 Table. Cord plasma PFAS concentrations among children with and without AD are reported in Table 3. Among the six PFASs, children with AD had higher PFOA concentration and lower PFUnDA concentration than those without AD.

**Table 2:** Concentration of the nine PFASs in cord plasma (ng/mL) (*N* = 839).

PFASs	GM (95%CI)	Mean (SD)	Median	IQR	Detection rate (%)
PFHxA	0.14 (0.13, 0.14)	0.15 (0.12)	0.13	0.13–0.13	3.33
PFHpA	0.15 (0.14, 0.15)	0.16 (0.10)	0.14	0.14–0.14	3.33
PFHxS	21.85 (20.81, 22.95)	25.21 (11.55)	23.00	16.95–32.05	99.29
PFOA	0.68 (0.63, 0.73)	1.19 (1.18)	0.65	0.23–1.96	50.66
PFNA	0.9 (0.8, 1.00)	3.27 (7.01)	0.93	0.36–2.58	87.00
PFOS	2.47 (2.25, 2.72)	4.24 (5.54)	3.48	2.18–5.05	89.27
PFDeA	0.2 (0.18, 0.21)	0.50 (1.11)	0.10	0.10–0.35	33.25
PFUnDA	0.65 (0.57, 0.74)	3.43 (7.24)	0.67	0.07–2.89	66.63
PFDoDA	0.18 (0.19, 0.16)	0.34 (0.37)	0.25	0.04–0.52	65.32

Abbreviations: AD, atopic dermatitis; GM, geometric mean; SD, standard deviation; IQR, interquartile range; PFAS, perfluoroalkyl and polyfluoroalkyl substance; PFHxA, perfluorohexanoic acid; PFHpA, perfluoroheptanoic acid; PFHxS, perfluorohexane sulfonic acid; PFOA, perfluorooctanoic acid; PFNA, perfluorononanoic acid; PFOS, perfluorooctane sulfonate; PFDeA: perfluorodecanoic acid; PFUnA, perfluoroundecanoic acid; PFDoDA, perfluorododecanoic acid

**Table 3:** Arithmetic mean [AM (SE)] of the six PFASs in cord plasma among children with and without atopic dermatitis (AD) (*N* = 839).

PFASs (ng/mL)	With AD (n = 45)		Without AD (n = 794)		p-value^¶^
	n	AM (SE)	n	AM (SE)	
PFHxS	45	26.99 (1.72)	794	25.11 (0.41)	0.273
PFOA	45	1.60 (0.18)	794	1.17 (0.04)	0.024
PFNA	45	3.07 (0.96)	792	3.14 (0.23)	0.071
PFOS	45	4.71 (0.48)	792	3.99 (0.12)	0.117
PFUnDA	44	1.32 (0.93)	792	3.31 (0.22)	0.003
PFDoA	45	0.27 (0.05)	794	0.35 (0.01)	0.092

^¶^Comparison of cord plasma PFAS levels for children with and without AD as analyzed by Kruskal-Wallis tests with outliers excluded.

Abbreviations: AD, atopic dermatitis; AM, arithmetic mean; SE, standard error; PFAS, perfluoroalkyl and polyfluoroalkyl substance; PFHxS, perfluorohexane sulfonic acid; PFOA, perfluorooctanoic acid; PFNA, perfluorononanoic acid; PFOS, perfluorooctane sulfonate; PFUnA, perfluoroundecanoic acid; PFDoDA, perfluorododecanoic acid.

We then grouped the PFOA concentrations into undetected and below and above the median in those with detected exposure. The other five PFASs including PFHxS, PFNA, PFOS, PFUA, and PFDoA were grouped by tertile. The associations between cord plasma PFAS concentrations and AD are shown in Table 4 after adjustment for sex, family income, maternal atopy, breast feeding, and maternal age during pregnancy. The results showed that children in the highest PFOA group had a significantly higher risk of developing AD (OR [95%CI] = 2.58 [1.27–5.32]) (Table 4). No significant association was found between the other five PFASs and AD in children at the age of 2 years (Table 4).

**Table 4:** Odds ratio (OR) for ever having AD in 2-year-old children according to in-utero exposure to PFASs (ng/mL) by simple and multiple regression analysis (*N* = 839).

PFASs	N	No. of AD (%)	OR (95% CI)	AOR (95% CI)^¶^
PFHxS				
< 19.05	280	11 (3.9)	Reference	Reference
19.05–28.55	280	17 (6.1)	1.58 (0.73, 3.54)	1.53 (0.68, 3.54)
≥28.55	279	17 (6.1)	1.59 (0.74, 3.55)	1.37 (0.60, 3.18)
PFOA				
< 0.46	414	18 (4.4)	Reference	Reference
0.46–1.96	213	7 (3.3)	0.75 (0.29, 1.75)	0.75 (0.26, 1.89)
≥1.96	212	20 (9.4)	2.29 (1.18, 4.47)*	2.58 (1.27, 5.32)**
PFNA				
< 0.51	282	20 (7.1)	Reference	Reference
0.51–1.84	279	16 (5.7)	0.80 (0.40, 1.57)	0.76 (0.36, 1.57)
≥1.84	278	9 (3.2)	0.44 (0.19, 0.95)*	0.44 (0.18, 1.01)
PFOS				
< 2.68	280	11 (3.9)	Reference	Reference
2.68–4.47	280	15 (5.4)	1.38 (0.63, 3.14)	1.33 (0.57, 3.20)
≥4.47	279	19 (6.8)	1.79 (0.85, 3.95)	1.86 (0.84, 4.36)
PFUnDA				
< 0.13	280	23 (8.2)	Reference	Reference
0.13–2.03	280	12 (4.3)	0.50 (0.24, 1.01)	0.60 (0.27, 1.28)
≥2.03	279	10 (3.6)	0.42 (0.19, 0.87)*	0.54 (0.23, 1.17)
PFDoDA				
< 0.05	291	19 (6.5)	Reference	Reference
0.05–0.41	271	15 (5.5)	0.84 (0.41, 1.68)	0.81 (0.38, 1.72)
≥0.41	277	11 (4.0)	0.59 (0.27, 1.25)	0.60 (0.26, 1.33)

^¶^Adjusted OR (AOR) was adjusted by sex, family income, maternal atopy, breast feeding, and maternal age at childbirth.

*P < 0.05

** *P* < 0.01.

Abbreviations: AD, atopic dermatitis; OR, odds ratio; CI, confident interval; AOR, adjusted OR; PFAS, perfluoroalkyl and polyfluoroalkyl substance; PFHxS, perfluorohexane sulfonic acid; PFOA, perfluorooctanoic acid; PFNA, perfluorononanoic acid; PFOS, perfluorooctane sulfonate; PFUnA, perfluoroundecanoic acid; PFDoDA, perfluorododecanoic acid.

We then stratified children by *GSTT1* and *M1* genotype and evaluated the joint effect of PFOA concentration and *GSTT1/M1* genotype on AD. After adjustment for sex, family income, maternal atopy, breast feeding, and maternal age during pregnancy, children with the *GSTT1*-null genotype that were in the highest PFOA group had a higher risk of developing AD (OR [95%CI] = 3.45 [1.26–9.99]). The result was similar for children with the *GSTM1*-null genotype (OR [95%CI] = 2.92 [1.12–7.91]) (Table 5).

**Table 5:** Relationship between PFOA Concentration (ng/mL) and ever having AD in 2-year-old children by regression analysis after stratification by *GSTT1/M1* genotype (*N* = 839).

Variable	N	No. of AD (%)	OR (95%CI)	AOR (95%CI)^¶^
***GSTT1*-null type**				
PFOA				
< 0.46	208	7 (3.4)	Reference	Reference
0.46–1.96	101	2 (2.0)	0.58 (0.09, 2.45)	0.61 (0.09, 2.65)
≥1.96	109	11 (10.1)	3.22 (1.23, 9.00)*	3.45 (1.26, 9.99)*
***GSTT1* present type**				
PFOA				
< 0.46	206	11 (5.3)	Reference	Reference
0.46–1.96	112	5 (4.5)	0.83 (0.26, 2.34)	0.85 (0.22, 2.81)
≥1.96	103	9 (8.8)	1.70 (0.66, 4.24)	1.83 (0.64, 5.21)
***GSTM1*-null type**				
PFOA				
< 0.46	234	11 (4.7)	Reference	Reference
0.46–1.96	120	4 (3.3)	0.70 (0.19, 2.09)	0.90 (0.23, 2.98)
≥1.96	118	11 (9.3)	2.08 (0.87, 5.02)^#^	2.92 (1.12, 7.91)*
***GSTM1* present type**				
PFOA				
< 0.46	180	7 (3.9)	Reference	Reference
0.46–1.96	93	3 (3.2)	0.82 (0.17, 3.04)	0.59 (0.09, 2.57)
≥1.96	94	9 (9.6)	2.62 (0.94, 7.55)	2.50 (0.84, 7.65)

^¶^AOR was adjusted by sex, family income, maternal atopy, breast feeding, and maternal age at childbirth.

^#^*P* < 0.1

*P < 0.05.

Abbreviations: AD, atopic dermatitis; OR, odds ratio; CI, confident interval; AOR, adjusted OR; PFOA, perfluorooctanoic acid; GST, glutathione s transferase.

## Discussion

To our knowledge, this is the first study to investigate the effect of PFAS exposure and *GST* genotype on childhood AD. We found that in-utero PFOA exposure was associated with AD development in 2-year-old children and this effect was more prominent among children carrying *GSTT1*-null or *GSTM1*-null genotypes.

Our result is consistent with the association between PFAS exposure and allergic diseases reported previously. In a prospective birth cohort study in China, prenatal exposure to PFOA, PFHxS, PFDoA, and PFOA significantly increased the risk of AD in girls at the age before 2 years old [24]. In a case–control study of Taiwanese children, asthmatic children exhibited significantly higher serum PFAS concentration than children without asthma [25]. PFOA is a common PFAS. Specifically, Anderson-Mahoney et al. indicated that residents with prolonged exposure to PFOA in drinking water had a higher prevalence of asthma than the general population in West Virginia, United States [26]. In the National Health and Nutrition Examination Survey (NHANES) study, PFOA was associated with ever having asthma among children at 12–19 years of age [27]. Notably, PFASs can cross the placental barrier, consequently accumulating in the foetus and affecting newborn health. In the present study, we found that cord serum PFOA was associated with childhood AD. This result was inconsistent with those of previous birth cohort studies in Japan and Taiwan. Okada et al. reported no association between maternal PFOA levels and eczema during the first 12 and 24 months [15]. Wang et al. found a positive correlation between cord blood IgE levels and cord serum PFOA in boys, although no association was found between PFOA exposure and AD in children [16]. This may have been due to insufficient samples for investigation (n = 244).

The biological mechanisms underlying the effects of PFAS exposure on AD development remain unclear. Allergen-specific responses in AD are skewed toward a Th2 phenotype with elevated serum IgE and IL-4 levels, whereas Fairley et al. reported that PFOA enhances the hypersensitivity response to ovalbumin with increasing IgE levels in a murine model [13]. Singh et al. found that PFOA triggers mast cell-derived allergic inflammatory reactions by histamine secretion and elevation of pro-inflammatory cytokines, including tumour necrosis factor alpha, IL-1, IL-6, and IL-8 [28]. Additionally, prenatal PFOA exposure was positively associated with cord blood IgE levels in a birth cohort study,[16] and a case–control study of children reported a dose–response effect of PFOA concentration on increasing IgE levels [14]. Stein et al. indicated that total IgE levels might increase by 10% in children with doubled PFOA exposure [29].

Another potential mechanism is through peroxisome proliferator-activated receptor (PPAR) signalling pathways. Both PPAR-α and PPAR-γ are potentially related to immune function owing to their expression on monocytes or macrophages [30]. PPARα agonists inhibit interferon-γ and enhance IL-4 levels [31]. PFOA is known as a peroxisome proliferator, and binding to PPAR-α increased the activation of mouse and human PPAR-α in an *in vitro* study [32]. Specifically, a dose–response effect was found for mouse PPAR-α activated by PFOA and a positive association was also found for human PPAR-α treated with PFOA. Moreover, this effect was inhibited by a PPAR-α antagonist [32]. PFOA may therefore potentially be related to the immune function of children and associated with AD development through a PPARα-activated pathway.

In our study, we also examined whether *GST* genetic variants comprised factors that modified the relationship of PFAS exposure to AD and found that PFOA exposure was associated with AD development, especially in children with *GSTT1*- or *GSTM1*-null genotypes. *GST* genotypes have also been previously reported to modify the effect of environmental exposure on allergic diseases. In a case–control study of Korean children, the *GSTM1*-null genotype was significantly associated with childhood AD onset [33]. However, Chung et al. found that a healthy dietary intake and the *GSTM1*-present genotype had a protective effect against AD [34]. Furthermore, Carlsten et al. indicated that the *GSTT1* genotype enhanced the ambient diesel exhaust exposure-mediated increase in allergen-sensitized inflammation in airways among atopic participants [35].

GSTs contribute to chemical detoxification by conjugation with glutathione, thereby protecting cells from reactive oxygen species (ROS). Oxidative stress is associated with the activation of inflammatory cells and production of pro-inflammatory cytokines and mediators [36]. ROS are known to associate with AD pathogenesis [37]. For example, a lower glutathione to glutathione disulphide ratio, representative of higher oxidative stress induction, was found in children with AD [38]. Additionally, PFOS and PFOA could induce ROS production and were associated with reductions in the antioxidative responses of hepatocytes, leading to oxidative damage [39]. Children with the *GSTT1-* or *GSTM1*-null genotype lose enzymatic activity and may therefore be vulnerable to the impacts of oxidative stress. Thus, the combination of PFOA exposure and *GSTT1/M1*-null genotypes is suspected to result in higher oxidative stress in children. Accordingly, we found that the effect of PFOA exposure on AD was more obvious in children with *GSTT1-* or *GSTM1*-null genotypes.

There are limitations in this study. The determination of childhood AD was based on maternal or main caregiver’s report of physician-diagnosed AD, which might result in misclassification. However, the validation of physician-diagnosed AD reported by mothers was confirmed by clinical examination in a previous study [40]. Moreover, characteristic symptoms of AD were described based on an international standard questionnaire, the ISAAC questionnaire. Children were identified as having AD based on both physician-diagnosed AD and a rash in a specific position, which might have reduced the level of misclassification. Second, approximately 33.6% of mother–newborn pairs were excluded from the final analysis owing to suspected cord blood contamination by maternal blood, loss of children to follow-up, and children without data for both PFAS and *GST* genotypes. Selection bias might thus be a concern. However, the characteristics of the children and mothers did not significantly differ between included and excluded children, aside from maternal education (S2 Table). Moreover, as the mothers and the interviewers were unaware of the research objective, selection bias caused by differential participation was less likely. Third, our studied newborns were recruited from eight private maternity hospitals located in seven areas. They may not be representative of all newborns in Taiwan. Caution about the generalizability of our findings is warranted. An external validation study in a more representative population is therefore warranted.

Despite these limitations, the study has several strengths. We enrolled a general population of pregnant women in their third trimester throughout Taiwan. Second, using a birth cohort design with longitudinal follow-up, we were able to clearly investigate the temporal sequence between early life environmental exposure and disease occurrence. Additionally, the prospective cohort design reduces recall bias. Finally, both PFAS concentrations and *GSTT1/M1* genotypes were measured using standard methods that therefore minimized the misclassification of these measurements.

In conclusion, **t**his study showed that in-utero PFOA exposure and neonatal *GSTT1* or *GSTM1* genotype might have joint effects and be associated with childhood AD. Avoiding and minimizing PFAS exposure in early life may be potentially helpful toward protecting against AD development.

## Supporting information

S1 Table Outlier points identified by Outlier analysis: Excluded data. (DOC)

S2 Table Characteristics of children and parents in included pairs (*N* = 839) and excluded pairs (*N* = 320). (DOC)

S3 Table Spearmen’s correlation between cord plasma PFAS concentrations (ng/mL) (*N* = 839). (DOC)

S1 Dataset (RAR)
