# Supplementary material for: Prenatal perfluorooctanoic acid exposure and glutathione s-transferase T1/M1 genotypes and their association with atopic dermatitis at 2 years of age
Source: PLoS One. 2019 Jan 16;14(1):e0210708. doi: 10.1371/journal.pone.0210708 (PMC6334968; doi:10.1371/journal.pone.0210708)
Supplement: S1 Table — (DOC) [file pone.0210708.s001.doc]

**S1 Table. Outlier points identified by Outlier analysis: excluded data**.

| PFASs | Value (ng/mL) |
| --- | --- |
| PFNA | 63 |
| PFNA | 54.5 |
| PFOS | 123.25 |
| PFOS | 56.5 |
| PFUA | 75 |
| PFUA | 64 |
| PFUA | 57.5 |

Abbreviations: PFAS, perfluoroalkyl and polyfluoroalkyl substance; PFNA, perfluorononanoic acid; PFOS, perfluorooctane sulfonate; PFUnA, perfluoroundecanoic acid.
