# Supplementary material for: Prenatal perfluorooctanoic acid exposure and glutathione s-transferase T1/M1 genotypes and their association with atopic dermatitis at 2 years of age
Source: PLoS One. 2019 Jan 16;14(1):e0210708. doi: 10.1371/journal.pone.0210708 (PMC6334968; doi:10.1371/journal.pone.0210708)
Supplement: S2 Table — (DOC) [file pone.0210708.s002.doc]

**S2 Table. Characteristics of children and parents in included pairs (*N* = 839) and excluded pairs (*N* = 320)**

^§^P value was calculated by Kruskal-Wallis tests for continues variables and χ2 test for categorical variables as compared between included pairs and excluded pairs.

| Characteristic | Included pairs | Excluded pairs | *P*-value^§^ |
| --- | --- | --- | --- |
|  | n (%) | n (%) |  |
| **Children** |  |  |  |
| Birth weight (g)^¶^ | 3104.6 (383.6) | 3075.9 (420.7) | 0.337 |
| Gestational length (weeks)^¶^ | 38.92 (1.20) | 38.81 (1.29) | 0.284 |
| Sex |  |  |  |
| Boy | 424 (50.5) | 163 (52.6) | 0.538 |
| Girl | 415 (49.5) | 147 (47.4) |  |
| Birth order |  |  |  |
| 1st | 358 (46.0) | 109 (41.9) | 0.438 |
| 2nd | 290 (37.3) | 108 (41.5) |  |
| ≥ 3rd | 130 (16.7) | 43 (16.6) |  |
| **Parents** |  |  |  |
| Maternal age at childbirth (year)^¶^ | 27.83 (4.69) | 27.48 (4.85) | 0.250 |
| Maternal education |  |  |  |
| ≤ 9 years | 84 (10.1) | 48 (15.3) | 0.010 |
| 10- 12 years | 474 (57.0) | 185 (58.9) |  |
| > 12 years | 274 (32.9) | 81 (25.8) |  |
| Paternal education |  |  |  |
| ≤ 9 years | 116 (14.0) | 51 (17.1) | 0.099 |
| 10–12 years | 435 (52.5) | 167 (55.9) |  |
| > 12 years | 277 (33.5) | 81 (27.1) |  |
| Family income (10^3^USD) |  |  |  |
| <$20 | 215 (27.2) | 90 (30.5) | 0.079 |
| $20−$40 | 287 (36.3) | 119 (40.3) |  |
| ≥$40 | 288 (36.5) | 86 (29.2) |  |
| Maternal atopy |  |  |  |
| Yes | 166 (19.8) | 43 (17.6) | 0.426 |
| No | 671 (80.2) | 202 (82.4) |  |
| Paternal atopy |  |  |  |
| Yes | 170 (20.3) | 44 (17.3) | 0.294 |
| No | 667 (79.7) | 210 (82.7) |  |

Some numbers do not add up to total *N* because of missing values.

^¶^mean (SD).

Abbreviations: USD, US dollars; SD, standard deviation.
