# Supplementary material for: Prenatal perfluorooctanoic acid exposure and glutathione s-transferase T1/M1 genotypes and their association with atopic dermatitis at 2 years of age
Source: PLoS One. 2019 Jan 16;14(1):e0210708. doi: 10.1371/journal.pone.0210708 (PMC6334968; doi:10.1371/journal.pone.0210708)
Supplement: S3 Table — (DOC) [file pone.0210708.s003.doc]

**S3 Table. Spearmen’s correlation between cord plasma PFAS concentrations (ng/mL) (*N* = 839)**

|  | PFHxS | PFOA | PFNA | PFOS | PFUnDA | PFDoA |
| --- | --- | --- | --- | --- | --- | --- |
| PFHxS | 1 |  |  |  |  |  |
| PFOA | -0.021 | 1 |  |  |  |  |
| PFNA | 0.139^***^ | -0.026 | 1 |  |  |  |
| PFOS | -0.022 | 0.374^***^ | 0.002 | 1 |  |  |
| PFUnDA | 0.088^*^ | -0.083^*^ | 0.686^***^ | -0.041 | 1 |  |
| PFDoA | 0.120^***^ | 0.012 | 0.739^***^ | -0.038 | 0.776^***^ | 1 |

^*^P<0.05, ^**^p<0.01, ^***^p<0.001.

Abbreviations: PFAS, perfluoroalkyl and polyfluoroalkyl substance; PFHxS, perfluorohexane sulfonic acid; PFOA, perfluorooctanoic acid; PFNA, perfluorononanoic acid; PFOS, perfluorooctane sulfonate; PFUnA, perfluoroundecanoic acid; PFDoDA, perfluorododecanoic acid.
